# Association Between the EAT-Lancet Diet Pattern and Risk of Type 2 Diabetes: A Prospective Cohort Study

**DOI:** 10.3389/fnut.2021.784018

**Published:** 2022-01-14

**Authors:** Chenjie Xu, Zhi Cao, Hongxi Yang, Yabing Hou, Xiaohe Wang, Yaogang Wang

**Affiliations:** ^1^School of Public Health, Hangzhou Normal University, Hangzhou, China; ^2^School of Public Health, Tianjin Medical University, Tianjin, China; ^3^Department of Big Data in Health Science, School of Public Health, Zhejiang University School of Medicine, Hangzhou, China; ^4^Department of Bioinformatics, School of Basic Medical Sciences, Tianjin Medical University, Tianjin, China

**Keywords:** EAT-Lancet diet pattern, type 2 diabetes, nutrition, UK biobank, metabolism

## Abstract

**Background:**

The EAT-Lancet Commission has promulgated a sustainable dietary guideline and recommended that it was designed to improve the human health and support environmental sustainability.

**Objective:**

This research was designed to explore the association between this healthy diet pattern (EAT-Lancet diet pattern, EAT-LDP) and risk of type 2 diabetes (T2D).

**Methods:**

Between 2006 and 2010, a total of 59,849 participants from the UK Biobank without diabetes, cardiovascular disease, or cancers were included at baseline. The EAT-LDP score was constructed on the sum of 14 food components and then categorized into three tertiles. Multivariable Cox proportional hazards regression models were conducted to explore the association between EAT-LDP score and the risk of incident T2D. A mediation analysis was also implemented to disentangle the role of body mass index (BMI) and waist circumference in the relationship between EAT-LDP score and T2D.

**Results:**

During a median follow-up of 10 years, 2,461 incident T2D cases were recorded. In analyses that compared tertile 3 of the EAT-LDP score (highest) with tertile 1 (lowest), the hazard ratio (HR) for T2D was 0.81 (95% CI: 0.72–0.90) after adjusting for sociodemographic status and health-related factors. Participants who reported a one-point increase in the diet score were associated with a 6% decrease in risk of T2D (HR: 0.94, 95% CI: 0.91–0.97). A significant indirect association was observed between the EAT-LDP score and T2D (β: 0.66, 95% CI: 0.65–0.67), indicating that 44% of the association of EAT-LDP score with T2D was mediated by BMI. Additionally, 40% of the association of EAT-LDP score with T2D was mediated by waist circumference was also observed.

**Conclusions:**

Our findings indicate that a higher adherence to EAT-LDP contributes to lower risk of T2D. Further independent validation is needed to be conducted before applying the EAT-LDP to inform dietary guidelines.

## Introduction

Diabetes mellitus, characterized by chronic hyperglycemia, refers to a metabolic condition that results from an interaction of genetic and environmental factors (1, 2). The prevalence of diabetes in the UK is up to 3.8 million people, accounting for about 9% of the adult population (3). Evidence has shown that suboptimal diet could be the driver of the obesity pandemic, which is the leading risk factor of preventable death and disability (4–6). Numerous studies have evaluated the associations between the specific food type and nutrient intake with type 2 diabetes (T2D) risk (7–9).

With the emphasis on overall diet quality, the diet pattern integrates potentially interactive and cumulative associations of different dietary components, which facilitate translation of findings into dietary recommendations. Diet patterns could reflect the numerous and multifaceted combinations of nutrient and food consumption in the real world (10). Multiple studies have found a beneficial effect of higher adherence to the plant-based Mediterranean (Medi) diet pattern on the risk of diabetes (11–13). In a comparative study, researchers concluded that both Healthy Eating Index (HEI) and Alternate Healthy Eating Index (AHEI) showed influences on T2D (14) in the US adults. A meta-analysis including 15 cohort studies showed that diets of the high quality are associated with a significant risk reduction for T2D and other chronic diseases (*p* < 0.05) (15). A prospective study among Taiwanese has found a protective effect of vegetarian diet on diabetes risk (16). The low levels of triglyceride to high-density lipoprotein cholesterol ratio diet pattern may reduce the incident T2D that has also been ascertained (17).

Here, we examined a new healthy and sustainable diet pattern, named EAT-Lancet diet pattern (EAT-LDP) raised by the EAT-Lancet Commission, which was designed to nurture human health and support environmental sustainability (18). It consists of whole grains, fruits, vegetables, legumes, nuts, unsaturated oils, low-to-moderate amount of seafood and poultry, and includes no or a low quantity of red meat, processed meat, added sugar, refined grains, and starchy vegetables (19). To date, few prospective studies have investigated the association between the EAT-LDP and adverse health conditions (20, 21). In this study, we further explored the association between EAT-LDP and incident T2D among the UK adults over a more than 10-year follow-up.

## Materials and Methods

### Data Source and Study Population

The UK Biobank is a large-scale biomedical database started from 2006. The aim of the program is to investigate the influence of genetic and environmental factors on disease development. It has recruited more than 500,000 volunteers aged 40 to 69 across the UK and will follow them over the next 30 years. Participants were invited to the assessment centers to complete a series of lifestyle, health and socioeconomic interviews, and physical measurements. In addition, biological samples were also collected. All disease conditions, prescription drug use, and deaths of them during the whole study period will be recorded through the centrally managed UK National Health Service system (22).

The initial 502,507 participants were invited to provide information on their food consumption in the past year through a touch questionnaire at the assessment centers. Subsequently, a sub-sample of about 20,000 population was also invited to repeat the questionnaire at assessment centers every 4 years, to examine the possible changes in participants' responses to the questionnaire and their dietary intake over time. Meanwhile, in order to gather more detailed information about the actual amount of food or food groups actually consumed by the participants, the UK Biobank also adopted an online 24-h dietary assessment tool named Oxford WebQ. It was developed for use in large population studies and had been validated in previous studies (23, 24). It collected additional detailed dietary intake information of 210,970 participants at least once through the 24-h recall. Participants were asked whether they have consumed the predefined 206 foods or 32 drinks in the past 24 h (24). After that, some of them were also invited to repeat the online 24-h questionnaire for a total of four times through emails between February 2011 and June 2012 every 3–4 months. At the same time, participants were also asked whether they consumed over the previous 24 h were fairly typical for their daily life (25).

**Figure 1:**
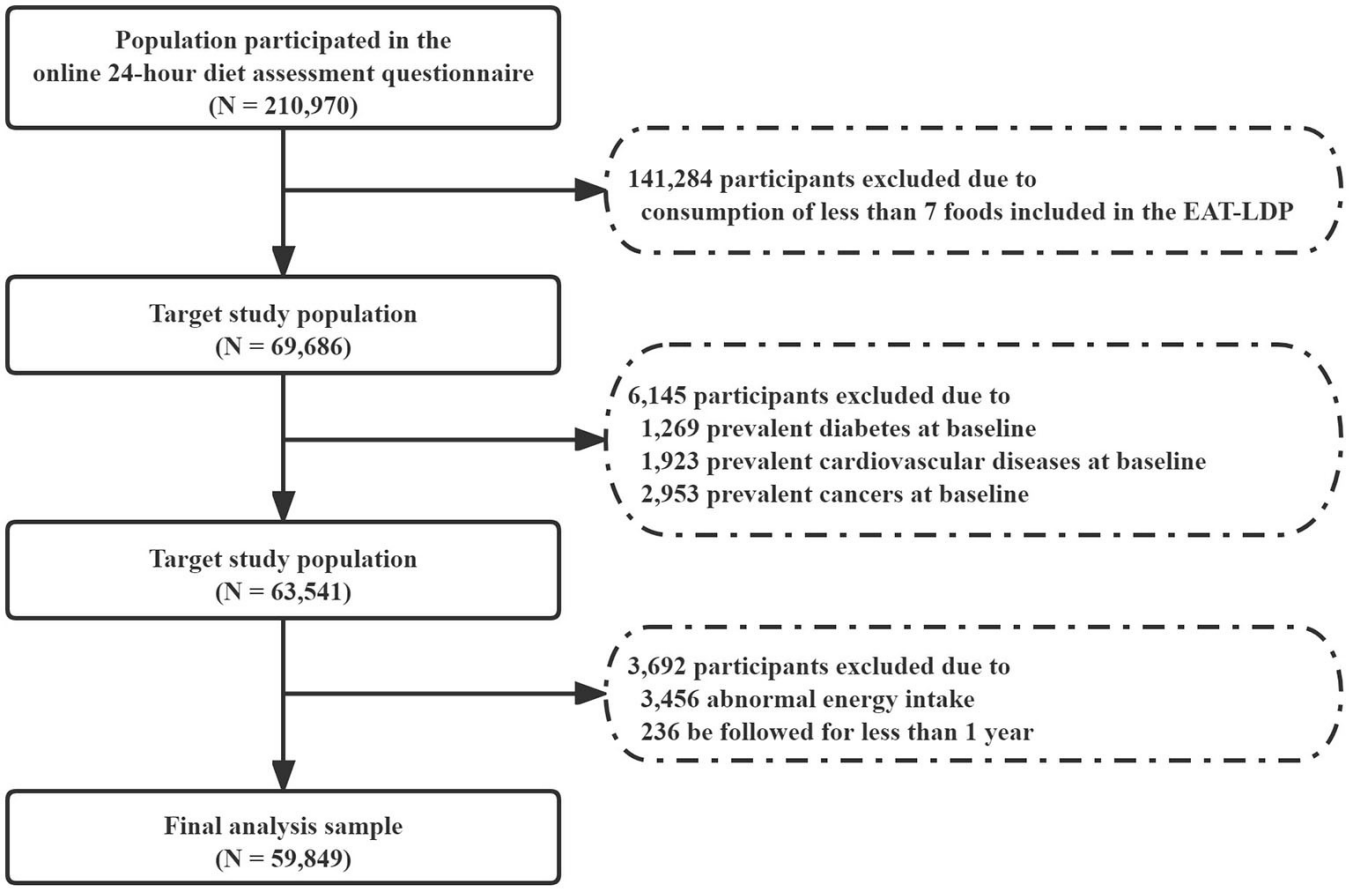
Flowchart of the research study design.

Participants who reported consumption of at least 7 foods included in the EAT-LDP based on the 24-h dietary assessment tool were included in the analysis first (*N* = 69,686). Later, who reported history of any cancer (2,953), cardiovascular disease (1,923), and diabetes (1,269) at the baseline were also excluded. We also excluded participants with abnormal total energy intakes (<2,093 or >14,650 kJ/day in female and <3,349 or >16,743 kJ/day in male participants). Finally, we excluded participants who were followed for <1 year (*N* = 236) to minimize the potential for reverse causality bias. As a result, a total of 59,849 participants were considered for inclusion in the following main analysis (Figure 1). All participants had given written informed consent.

### Definition of EAT-LDP Score

The EAT-LDP score used in this study was designed by Knuppel et al. (20). The distribution of the diet score can be seen in Supplementary Table 1. Definition of portion size and food items used in this study can be seen in Supplementary Table 2. The EAT-LDP score is consisted of 8 main dietary components, including whole grains, tubers and starchy vegetables, vegetables, fruits, dairy foods, protein sources, added fats, and added sugars. Participants were assigned with a point for meeting each of the recommendations. Each dietary component contributed 0 or 1 point resulting in a total score ranging from 0 to 14 points. The higher dietary scores indicated a greater adherence to the individual healthy eating patterns.

The online 24-h dietary assessment tool did not record the concrete weight of consumed food, but the number of predefined portion size was defined using the UK's standard food composition database (26, 27) (e.g., how many bowls of cereals, how many serving of Quorn they ate in the last 24 h, Supplementary Table 2). We then calculated the quantity by multiplying the portion size by the number of portions consumed for each food item.

### Assessment of Type 2 Diabetes

The primary outcome of this study was the incident cases of T2D. The T2D diagnosis during follow-up was ascertained according to the International Classification of Diseases, 10th edition (ICD-10) (28) code from the hospital where the inpatient records containing data on admissions and diagnoses linked to the Hospital Episode Statistics for England, Scottish Morbidity Record data for Scotland, and the Patient Episode Database for Wales. Record linkage was available until March 2020.

### Assessment of Covariates

Sociodemographic characteristics and lifestyle factors were recorded at baseline, including age, sex, ethnicity (White, Asian or Asian British, Black or Black background, Chinese, mixed background, etc.), Townsend deprivation index (an index constructed based on the material deprivation degree) (29), education attainment (college or university degree, professional qualifications, etc.), smoking status (previous, current, and never), drinking status (previous, current, and never), body mass index (BMI, kg/m^2^), waist circumference (cm), total energy intake (kJ/day), and metabolic equivalent (MET; min/week for all activity including walking, and moderate and vigorous activity). A separate category was introduced for those who had no available data on smoking (164 missing) or drinking (64 missing) status.

### Statistical Analysis

We used mean and SD to express normally distributed continuous variables, and number (percentages) to express categorical variables. Baseline characteristics are summarized and compared by using the χ^2^ test for categorical, or ANOVA or Mann–Whitney *U-*test for continuous variables, as appropriate. If covariate information was missing, we imputed the mean values for continuous variables or used a missing indicator approach for categorical variables.

We calculated each participant's person-years from the date of the return of the baseline questionnaire (2006–2010) to the date of diabetes diagnosis, death, loss to follow-up, or the end of follow-up (March 31, 2020), whichever came first. Incidence rates of T2D events per 1,000 person-years were calculated by the EAT-LDP score. We used Cox proportional hazards models to examine the association between the diet score and the subsequent risk of T2D. The proportional hazards assumption was tested with Schoenfeld residuals against survival time.

We divided the study participants into 3 groups according to their summed EAT-LDP scores and compared the HRs with Tertile 1 as the reference group. The cut-offs for the groups were chosen so that the number of participants in each group is reasonable and similar within the allowable range of data distribution (Supplementary Figure 1). Meanwhile, the mean food intakes at baseline were calculated from the participants within each group who had reported consuming the specific food component.

Four models were estimated, and covariates were added in a stepwise manner. The Model 1 was the crude model. In Model 2, we further adjusted for sociodemographic covariates, including age sex, deprivation, qualifications, and ethnicity. In Model 3, we additionally adjusted for lifestyle factors included smoking, drinking, energy intake, and MET. And Model 4 additionally included baseline BMI and waist circumference. In addition, we explored the potential non-linear associations using 3-knotted restricted cubic spline regression. We also viewed the diet score as a linear variable to examine the risk reduction associated with a 1-point increment.

A mediation analysis was considered to explicate the association of diet score with T2D; indirect associations acting through BMI as a mediating variable and direct associations not mediated by BMI were quantified. At the same time, a mediation analysis was also conducted when waist circumference was included in the Cox models. We used *paramed* package in Stata version 16 (StataCorp., College Station, TX, USA) to conduct the mediation analysis. This is a parametric regression approach that estimates two models. For example, one model for the mediator (BMI) conditional on exposure (diet score) and covariates, and another model for the T2D conditional on diet score, BMI and covariates. It extends statistical mediation analysis to allow for the presence of exposure-mediator (diet score and BMI) interactions in the outcome regression model using counterfactual definitions of direct and indirect effects. The proportion of the association mediated by BMI or waist circumference was calculated by the formula: NIE/(NIE + NDE) (32). NIE represents the natural indirect effects, and NDE represents the natural direct effects of EAT-LDP score on T2D. We also used *testparm* package to test the statistical significance of potential interaction between the exposure variable and covariates (30). The *p-*values for interaction were evaluated using interaction terms and likelihood ratio tests.

Several sensitivity analyses were performed to test the robustness of our findings as follows. First, we conducted stratified analyses that were defined a priori by ethnicity and other potential risk modifiers, including age, sex, deprivation, education attainment, smoking, and drinking status. Second, we repeated all analyses after excluding all participants who developed T2D during the first 3 years of follow up to reduce the possibility of spurious association due to reverse causation. Third, we conducted the multiple imputation (*mi* package) to impute the missing covariates, and 5 imputed datasets were pooled using Rubin's rules. The statistical analysis was conducted using Stata version 16 (StataCorp., College Station, TX, USA). The statistical significance was set as *p* < 0.05 (two-sided test).

## Results

### Characteristics of the Included Participants

We described the baseline characteristics of the included participants according to the tertiles of EAT-LDP score in Table 1. A total of 59,849 participants were included in the analyses, with an average age of 55.9 years. Among them, there are 34,512 (57.67%) female and 25,337 (42.33%) male participants. The mean dietary score among these targeted participants was 5 (range from 1 to 10). Participants with higher EAT-LDP scores tended to be older [mean (SD) age, 56.96 (7.89), *p* < 0.001], female (63.92%, *p* < 0.001), white (94.94%, *p* < 0.001), be least deprived (21.79%, *p* < 0.001), have other education attainment (51.22%, *p* < 0.001), be never smokers (60.84%, *p* < 0.001), with lower BMI [mean (SD), 26.36 (4.44), *p* < 0.001], smaller waist circumference [mean (SD), 86.66 (12.65), *p* < 0.001], and have higher total physical activity [PA, mean (SD), 2,756.06 (2547.09), *p* < 0.001]. Overall, the group with the lowest diet score had the highest incidence rate of T2D (Table 2).

**Table 1:** Sociodemographic characteristics of the study population by the EAT-LDP score group.

**Characteristic**	**Total**	**Tertiles of EAT-LDP scor**			***P*-value**
		**T1**	**T2**	**T3**	
**Total**, ***n*** **(%)**	59,849	27,527 (45.99)	15,188 (25.38)	17,137 (28.63)	
**Total energy intake (kJ/day), mean (SD)**	8,463.00 (2,442.31)	8,218.17 (2,517.47)	8,547.62 (2,400.66)	8,781.33 (2,311.17)	<0.001
**Age (years), mean (SD)**	55.91 (8.14)	55.08 (8.24)	56.21 (8.09)	56.96 (7.89)	<0.001
**Sex**, ***n*** **(%)**					<0.001
Female	34,512 (57.67)	14,547 (52.85)	9,013 (59.34)	10,952 (63.92)	
Male	25,337 (42.33)	12,980 (47.15)	6,175 (40.66)	6,182 (36.08)	
**Ethnicity**, ***n*** **(%)**					<0.001
White	56,049 (93.65)	25,420 (92.35)	14,362 (94.56)	16,267 (94.94)	
Asian or Asian British	452 (0.76)	226 (0.82)	105 (0.69)	121 (0.71)	
Black or black background	1,284 (2.15)	674 (2.45)	276 (1.82)	334 (1.95)	
Chinese	1,114 (1.86)	709 (2.58)	214 (1.41)	191 (1.11)	
Mixed background	176 (0.29)	93 (0.34)	36 (0.24)	47 (0.27)	
Others	774 (1.29)	405 (1.47)	195 (1.28)	174 (1.02)	
**Townsend deprivation index**, ***n*** **(%)**					<0.001
1 (least deprived)	11,972 (20.04)	5,048 (18.37)	3,196 (21.08)	3,728 (21.79)	
2	11,964 (20.05)	5,300 (19.29)	3,044 (20.07)	3,620 (21.16)	
3	11,944 (19.99)	5,493 (19.99)	3,034 (20.01)	3,417 (19.97)	
4	11932 (19.97)	5475 (19.93)	3072 (20.26)	3385 (19.78)	
5 (Most deprived)	11,936 (19.98)	6,159 (22.42)	2,818 (18.58)	2,959 (17.29)	
**Education attainment**, ***n*** **(%)**					<0.001
College or university degree	23,617 (39.46)	9,846 (35.77)	6,314 (41.57)	7,457 (43.52)	
Professional qualifications	2,894 (4.84)	1,234 (4.48)	759 (5.00)	901 (5.26)	
Others	33,338 (55.70)	16,447 (59.75)	8,115 (53.43)	8,776 (51.22)	
**Smoking status**, ***n*** **(%)**					<0.001
Never	34,432 (57.53)	15,089 (55.00)	8,940 (59.01)	10,403 (60.84)	
Previous	20,369 (34.13)	9,416 (34.32)	5,145 (33.96)	5,808 (33.96)	
Current	4,884 (8.18)	2,930 (10.68)	1,065 (7.03)	889 (5.20)	
**Drinking status**, ***n*** **(%)**					0.005
Never	2,194 (3.67)	1,020 (3.71)	558 (3.67)	616 (3.60)	
Previous	1,891 (3.16)	939 (3.41)	457 (3.01)	495 (2.89)	
Current	55,700 (93.07)	25,535 (92.76)	14,151 (93.17)	16,014 (93.46)	
**Obesity-related markers**
BMI, mean (SD)	26.97 (4.64)	27.43 (4.74)	26.85 (4.60)	26.36 (4.44)	<0.001
Waist circumference	88.77 (13.20)	90.33 (13.37)	88.34 (13.14)	86.66 (12.65)	
Total PA (MET-min/week), mean (SD)	2,668.23 (2,604.84)	2,601.35 (2,643.52)	2,687.34 (2,597.38)	2,756.06 (2,547.09)	<0.001

*BMI, body mass index; EAT-LDP, EAT-Lancet diet pattern; PA, physical activity; MET, metabolic equivalent*.

**Table 2:** HRs (95% CIs) for the associations between EAT-LDP score and incidence of T2D (*n* = 59,849).

	**No. of participants (%)**	**Cases of T2D**	**Incidence rate per 1,000 person-year (95% CI)**	**Model 1^a^**	**Model 2^b^**	**Model 3^c^**	**Model 4^d^**
**Tertiles of EAT-LDP score**							
T1	27,527 (45.99)	1,302	4.74 (4.49, 5.00)	1.00 (reference)	1.00 (reference)	1.00 (reference)	1.00 (reference)
T2	15,188 (25.38)	567	3.72 (3.43, 4.04)	0.79 (0.71, 0.87)	0.82 (0.75, 0.91)	0.85 (0.76, 0.95)	0.90 (0.81, 1.01)
T3	10,986 (18.36)	592	3.44 (3.17, 3.73)	0.72 (0.66, 0.80)	0.77 (0.70, 0.85)	0.81 (0.72, 0.90)	0.95 (0.81, 1.06)
*P* for trend				<0.0001	<0.0001	<0.0001	0.249
1-point increment in diet score	59,849	2,461	4.11 (3.95, 4.27)	0.90 (0.88, 0.93)	0.92 (0.90, 0.95)	0.94 (0.91, 0.97)	0.99 (0.96, 1.02)

*HRs, hazard ratios; EAT-LDP, the EAT-Lancet diet pattern; T2D, type 2 diabetes*.

a *Crude model*.

b *Adjusted for age, sex, Townsend deprivation index, qualifications, and ethnicity*.

c *Adjusted for age, sex, Townsend deprivation index, qualifications, ethnicity, smoking, drinking, physical activity, and energy*.

d *Adjusted for age, sex, Townsend deprivation index, qualifications, ethnicity, smoking, drinking, physical activity, energy, BMI, and waist circumference*.

The proportion of target population adhering to each diet component is shown in Supplementary Table 3. The mean intake for each food component (for whole population and in each tertile) at baseline is also shown in Supplementary Table 4. In this study, 82% of the 24-h diet recalls were considered to reflect typical eating habits. Of the 59,849 selected participants finally included in the analysis, more than 50% of them consumed the recommended weights of whole grains and refined grains, all vegetables, all fruits, dairy foods, eggs, and soy foods. Participants with the highest EAT-LDP scores (3rd tertile) were more likely to consume the recommended weights of potatoes, all vegetables, dairy foods, protein foods, legumes, added fats, and added sugars.

### EAT-LDP Score and Incident T2D

Results of the associations between the EAT-LDP adherence and risk for T2D are shown in Table 2. During a mean follow-up time of 10 years, a total of 2,461 adults experienced the incident T2D (4.11 per 1,000 person-years, 95% CI: 3.95–4.27). Across tertiles of the EAT-LDP score (from lowest to highest), the incidence of T2D decreased from 4.74 to 3.44 per 1,000 person-years. All models, except the Model 4 adjusted for BMI and waist circumference, consistently showed a significant dose–response relationship between the increased diet score and risk of T2D. In multivariate analysis (Model 3), compared with the group with the lowest diet score (1st tertile), the HR for T2D was 0.81 (0.72–0.90).

**Figure 2:**
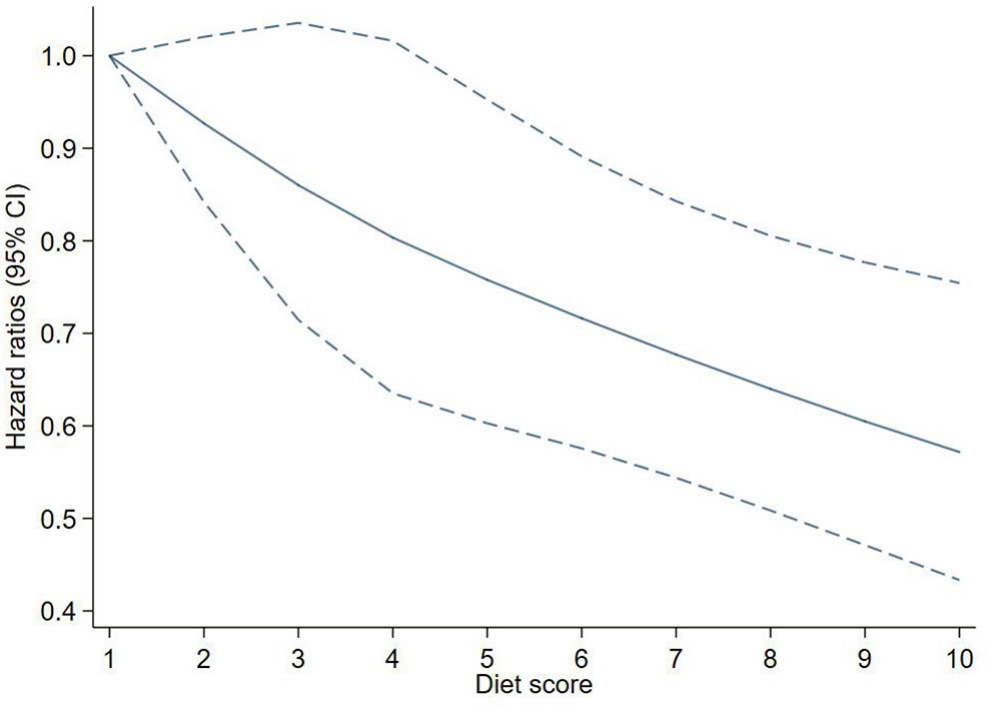
Adjusted hazard ratios (HRs) of T2D events risk according to EAT-LDP score. The figure shows HRs for T2D adjusted for age, sex, Townsend deprivation index, qualifications, ethnicity, smoking, drinking, physical activity, energy, BMI, and waist circumference. BMI, body mass index; EAT-LDP, the EAT-Lancet diet pattern; T2D, type 2 diabetes.

After adjusting for potential confounders, each additional point of the EAT-Lancet diet score was associated with a 6% decreased risk of T2D (HR: 0.94, 95% CI: 0.91–0.97). A linear association between the diet score and incident T2D using restricted cubic spline regression can be found in Figure 2.

Supplementary Figure 2 shows the association between the adherence of specific food recommendations and incident T2D. We found that adhering to the recommended intake of potatoes, all vegetables, all fruits, dairy foods, beef, lamb and pork, and eggs was associated with lower incidence of T2D in this analysis. In our research, 914 participants met the recommendation ( ≤ 31 g/day) of all sugars. The result shows that the consumption of all sugars was significantly associated with the T2D risk (HR = 1.85, 95% CI: 1.45–2.37). Considering that the purpose of this research is to explore the relationship between the specific diet pattern and T2D, a single-diet component is not the focus of this study.

### Mediation Analysis

Table 3 presents the total, direct, and indirect associations between the EAT-LDP score and T2D, and 44% of this association was mediated by BMI as mediator variable. The total effect was significant (β = 0.56, 0.54–0.57, *p* < 0.0001), with the natural direct effects (β = 0.85, 0.82–0.88, *p* < 0.0001) and the natural indirect effect (β = 0.66, 0.65–0.67, *p* < 0.0001). Similar results were found within sex-stratified analyses. The relationship between the EAT-LDP score and risk of T2D was 36% mediated by BMI among male and 53% among female participants.

**Table 3:** Adjusted direct and indirect associations of T2D with EAT-LDP score mediated *via* BMI, and waist circumference.

	**Overall (*****N*** = **59,849)**		**Male (*****N*** = **25,337)**		**Female (*****N*** = **34,512)**	
**Measures**	**β (95% CI)**	***P-* value**	**β (95% CI)**	***P-*value**	**β (95% CI)**	***P-*value**
**BMI**
Marginal total association	0.56 (0.54, 0.57)	<0.0001	0.70 (0.67, 0.73)	<0.0001	0.42 (0.40, 0.44)	<0.0001
Natural direct association	0.85 (0.82, 0.88)	<0.0001	1.12 (1.07, 1.17)	<0.0001	0.61 (0.58, 0.65)	<0.0001
Natural indirect association *via* BMI	0.66 (0.65, 0.67)	<0.0001	0.62 (0.61, 0.63)	<0.0001	0.68 (0.67, 0.69)	<0.0001
Proportion mediated (%)	44	36	53
**Waist circumference**						
Marginal total association	0.55 (0.53, 0.56)	<0.0001	0.69 (0.66, 0.72)	<0.0001	0.41 (0.39, 0.43)	<0.0001
Natural direct association	0.90 (0.87, 0.93)	<0.0001	1.13 (1.08, 1.18)	0.020	0.63 (0.61, 0.67)	<0.0001
Natural indirect association *via* waist circumference	0.61 (0.53, 0.56)	<0.0001	0.61 (0.60, 0.62)	<0.0001	0.64 (0.63, 0.65)	<0.0001
Proportion mediated (%)	40	35	50

*BMI, body mass index; EAT-LDP, the EAT-Lancet diet pattern; T2D, type 2 diabetes. Adjusted for age, sex, Townsend deprivation index, qualifications, ethnicity, smoking, drinking, physical activity, and energy*.

Table 3 also shows that the mediating role of the waist circumference in the association between the EAT-LDP score and incident of T2D (40%). Similarly, the relationship between the EAT-LDP score and risk of T2D was 35% mediated by waist circumference among male and 50% among female participants.

### Sensitivity Analysis

We observed similar results in the analysis that included participants who were followed for <3 years, which reinforced the robustness of our findings (Supplementary Table 5). The results did not significantly change when the multiple imputation was conducted (Supplementary Table 6). We also explored the associations between the EAT-LDP score and incident T2D stratified by age, sex, ethnicity, Townsend deprivation index, education attainment, smoking status, and drinking status. In the stratified analysis, compared tertile 3 of the EAT-LDP score with tertile 1, the hazard ratio (HR) for T2D in female participants was 0.72 (95% CI: 0.61–0.86). The protective effects of adherence to higher EAT-LDP score can be seen in the white, never and previous smoking, and current drinking participants (Supplementary Table 7). Meanwhile, the effect modification by these covariates is not significant.

## Discussion

This study explored the association between a healthy diet score and risk of T2D in a large population of 59,849 middle-aged adults in the UK. During a median 10 years of follow-up, 2,461 participants developed T2D. Our results show that greater adherence to EAT-LDP was associated with lower T2D risk over time.

Our findings appear to be consistent with the previous studies (20, 31), reporting the EAT-LDP shows beneficial associations for diabetes. With data from the EPIC-Oxford study, Knuppel et al. simultaneously investigated the associations of the EAT-LDP with major health outcomes (including ischemic heart disease, stroke, diabetes, and all-cause mortality) (20). They have drawn the conclusion that the EAT-Lancet reference diet shows beneficial associations for incidence of ischemic heart disease and diabetes, which is consistent with our research. Every individual component of the EAT-LDP has been separately investigated in this study. According to our exploratory results, the adherence to the recommended intake of potato, vegetables, fruits, dairy foods, beef, lamb, and pork were associated with lower T2D incident risk. The specific food components, like fruits, vegetables, legumes, olive oil and fish, have been verified to be associated with better health status (32–34). Consumption of these foods are related to lower body weight, hemoglobin A1c, low-density lipoprotein (LDL) and oxidative stress, and improved high-density lipoprotein (HDL), which are beneficial to the improvement of the prevention and prognosis of T2D (35, 36). Some specific food components were not associated with T2D risk, and this might indicate that the synergistic effects that occur in the EAT-LDP bring superior benefits compared with those from each isolated nutrition (37). Our result also shows that not adhering to the recommendation of the added sugars (≤ 31 g/day) was associated with a greater risk for T2D. It has been assumed that excess sugar can promote weight gain through extra calories intake, thus T2D (38). In 2015, based on 17 cohorts prespecified information, Imamura et al. concluded that the habitual consumption of sugar sweetened beverages was associated with a greater incidence of T2D (39).

Adhering to the recommendation amount of grains (≤ 464 g/day) was not associated with T2D risk (*p* > 0.675), which was not consistent with previous studies (40, 41), as both whole grain and refined grains were included in this research. Previous study has found an increased risk of colorectal cancer in those with high intakes of red and processed meat (42). Our study found that people who were consuming an average of ≤ 28 g/day beef, lamb, and pork had a 40% (95% CI: 0.40–0.90) lower risk of T2D. This means higher beef intake is associated with increased T2D risk. Proportion of the study population adhering to the protein foods is relatively small. Currently, recommendations of protein intake are based on individual assessment and the consideration of health issues (43). We need to further explore the underlying associations between the specific food component and risk of the T2D.

Much of the existing literature has considered obesity indicators such as BMI, waist circumference, or waist–hip ratio (WHR) as confounders and adjusted them in the Cox model (44, 45). However, we conducted the mediation analysis and observed that the association between the EAT-Lancet diet adherence and the risk of T2D was 44% mediated by BMI, or 40% by waist circumference. We also observed a direct effect of the healthy diet, suggesting that EAT-LDP can prevent T2D even if it does not lead to change to BMI or waist circumference. Our results have strong biological plausibility. Laouali et al. found that a higher anti-inflammatory potential of the diet is associated with a lower risk of T2D with BMI as a mediator factor in a France population (46). Fan et al. prospectively followed 10,419 Chinese adults and concluded that the waist circumference and its change were strongly associated with the risk of T2D (47). Previous studies have observed that weight loss among overweight or obese patients with T2D was consistently associated with a reduction of hemoglobin A1c, insulin resistance, and leptin levels, which involved in the pathogenesis of T2D (48, 49).

### Strengths and Limitations

This study has the advantages of prospective design and large sample size of diet habits to explore the association between the healthy and sustainable EAT-LDP and incident of T2D. Our results of the main analysis are shown to be consistent with the sub-analyses. There are some potential limitations warrant consideration. First, each component of EAT-LDP score was constructed as a binary variable (adherence to the target intake levels vs. non-adherence). This may lead to the loss of some dietary information. A more refined scoring method should be developed to investigate its association with the possible health status. Second, participants in our study can only represent middle-aged and elderly people. And 93% of people are white. Target participants included in our main analysis were those who reported consumption of at least 7 foods according to the EAT-LDP. Therefore, the obtained results could not be generalized to other population with different characteristics. At the same time, the dietary information used in this analysis mainly comes from the baseline assessment, which may not reflect the potential changes in participants' eating habits. Third, patients diagnosed early were followed for a longer period of time than patients diagnosed in recent years. Longer follow-up time would allow the increase of the duration between nutritional assessment and assessment of the T2D. Fourth, T2D in this research was diagnosed through inpatient medical records. Although doctors' diagnosis is a more common and precise way, the actual incidence of T2D could be underestimated. Last but not least, although we have adjusted for different confounding factors, there may be residual of unmeasured confounding factors that cannot be excluded in the observational studies. More validation is needed for reliable estimation of the associations between EAT-LDP and the possible adverse health outcomes.

### Conclusions

In light of the increasing global burden of diabetes, our results seem to be clinically relevant for diabetes prevention, and the EAT-LDP is an achievable and sustainable objective that should be promoted.

## Data Availability Statement

Details of the UKB data are available upon reasonable request (https://bbams.ndph.ox.ac.uk/ams/resApplications).

## Ethics Statement

The studies involving human participants were reviewed and approved by NHS National Research Ethics Service (NW/0382). The patients/participants provided their written informed consent to participate in this study.

## Author Contributions

YW contributed to the conception and ideas. YW, XW, CX, and ZC contributed to the study design. YW and ZC had full access to all of the data in the study. CX and ZC performed the statistical analysis, results interpretation, and assisted by HY and YH. CX and ZC wrote the first and successive drafts of the manuscript. All authors contributed to the article and approved the submitted version.

## Funding

This work was supported by National Natural Science Foundation of China (Grant No. 91746205) and Scientific Research Foundation for Scholars of HZNU (Grant No. 4265C50221204119 to CX).

## Conflict of Interest

The authors declare that the research was conducted in the absence of any commercial or financial relationships that could be construed as a potential conflict of interest.

## Publisher's Note

All claims expressed in this article are solely those of the authors and do not necessarily represent those of their affiliated organizations, or those of the publisher, the editors and the reviewers. Any product that may be evaluated in this article, or claim that may be made by its manufacturer, is not guaranteed or endorsed by the publisher.

## Abbreviations

EAT-LDP: the EAT-Lancet diet pattern
T2D: type 2 diabetes
BMI: body mass index
PA: physical activity
MET: metabolic equivalent
HR: hazard ratio.
