# Supplementary material for: Association Between the EAT-Lancet Diet Pattern and Risk of Type 2 Diabetes: A Prospective Cohort Study
Source: Front Nutr. 2022 Jan 14;8:784018. doi: 10.3389/fnut.2021.784018 (PMC8795697; doi:10.3389/fnut.2021.784018)
Supplement: Supplementary file 1 [file Data_Sheet_1.pdf]

Supplementary Table 1: Distribution of EAT-LDP score.

**Component**	**Criteria for minimum score of 0**	**Criteria for maximum score of 1***
Whole grains and refined grains		
1 Rice, wheat, corn, and other	>464 g/day	≤ 464 g/day
Tubers and starchy vegetables		
2 Potatoes	>100 g/day	≤100 g/day
Vegetables		
3 All vegetables	<200 g/day	≥200 g/day
Fruits		
4 All fruits	<100 g/day	≥100 g/day
Dairy foods		
5 Whole milk or derivative equivalents	>500g/day	≤500 g/day
Protein sources		
6 Beef, lamb, pork	>28 g/day	≤28 g/day
7 Chicken, other poultry	>58 g/day	≤58 g/day
8 Eggs	>25 g/day	≤25 g/day
9 Fish	>100g/day	≤100g/day
Legumes		
10 Dry beans, lentils, peas	>100 g/day	≤100g/day
11 Soy foods	>50 g/day	≤50g/day
12 Peanuts or tree nuts	<25 g/day	≥25g/day
Added fats		
13 Palm oil, unsaturated oils, dairy fats (incl. in milk), lard or tallow		Ratio of 0.8 for unsaturated: saturated fat intake**
Added sugars		
14 All sweeteners	> 31g/day	≤ 31g/day

*Each food component contributed 1 point if participants met the recommendation of the EAT-Lancet diet pattern.

**Too little information about the types of oils were recorded in the 24-hour diet recall so fat intake was approximated as a ratio of total unsaturated and saturated fat in line with the EAT-Lancet reference diet [Reference 20].

†Derived from sweet foods and beverages, and sugar added to food or beverages recorded in the 24-hour diet recall.

Supplementary Table 2: Definition of portion size and food items used in this study.

Component	Definition of 1 portion	Food items based on the Oxford WebQ questionnaire (24-hour dietary assessment tool)
Whole grains and refined grains		
1 Rice, wheat, corn, and other	1 portion: 130 g	porridge, muesli, oat crunch, sweetened cereal, plain cereal, bran cereal, whole-wheat cereal, other cereal, white pasta, wholemeal pasta, white rice, brown rice, sushi, snackpot, couscous, other grain
Tubers and starchy vegetables		
2 Potatoes	1 portion: 58 g	Fried, boiled, baked or mashed potatoes, sweet potato
Vegetables		
3 All vegetables	1 portion: 100 g	quorn, mixed vegetable, vegetable pieces, coleslaw, side salad, avocado, broad bean, green bean, beetroot, broccoli, butternut squash, cabbage, carrot, cauliflower, celery, courgette, cucumber, garlic, leek, lettuce, mushroom, onion, parsnip, pea, sweet pepper, spinach, fresh tomato, tinned tomato, turnip, watercress, other vegetable, sweetcorn
Fruits		
4 All fruits	1 portion: 150 g	stewed fruit, prune (1 portion: 48g), dried fruit (1 portion: 48g), mixed fruit, apple, banana, berry (1 portion: 48g), cherry (1 portion: 48g), grapefruit, grape (1 portion: 48g), mango, melon, orange, satsuma, peach, pear, pineapple, plum (1 portion: 48g), other fruit
Dairy foods		
5 Whole milk or derivative equivalents	1 portion: 258 g	milk, favoured milk, yogurt (1 portion: 180g), low fat hard cheese, hard cheese, soft cheese, blue cheese, low fat cheese spread, cheese spread, cottage cheese, feta, mozzarella, goat's cheese , other cheese (1 portion: 28g)
Protein sources		
6 Beef, lamb, pork		bacon, ham (1 portion：40g), liver, sausage, beef, pork, lamb (1 portion：80g)
7 Chicken, other poultry	1 portion：80 g	crumbed or deep-fried poultry, poultry
8 Eggs	1 portion：60 g	whole egg, omelette, eggs in sandwich, scotch egg, other egg
9 Fish	1 portion: 100 g	shellfish, tinned tuna, oily fish, breaded fish, battered fish, white fish, other fish
Legumes		
10 Dry beans, lentils, peas	1 portion: 100 g	dried lentils, peas and baked beans
11 Soy foods	1 portion: 100 g	tofu
12 Peanuts or tree nuts	1 portion: 28 g	unsalted peanuts, salted nuts, unsalted nuts, seed
Added fats		
13 Palm oil, unsaturated oils, dairy fats (incl. in milk), lard or tallow		Ratio of 0.8 for unsaturated: saturated fat intake*

Added sugars		
14 All sweeteners†		derived from sweet foods and beverages, and sugar added to food or beverages recorded in the 24-hour diet recall

*Too little information about the types of oils were recorded in the 24-hour diet recall so fat intake was approximated as a ratio of total unsaturated and saturated fat in line with the EAT-Lancet reference diet [Reference 20].

†Derived from sweet foods and beverages, and sugar added to food or beverages recorded in the 24-hour diet recall.

Supplementary Table 3: Numbers and proportions of the participants adhering to the recommended food intake by EAT-LDP score.

Component	Total numbers of participants, n(%)*	Tertiless of EAT-LDP score			*P* value
		T1	T2	T3	
**Total, n(%)**	59849	27527 (45.99)	15188 (25.38)	17137 (28.63)	
**Whole grains *and refined grains*, n(%)**					
1 Rice, wheat, corn, and other	45100 (75.36)	17300 (62.85)	12301 (80.99)	15523 (90.58)	<0.001
**Tubers and starchy vegetables, n(%)**					
2 Potatoes	27208 (45.46)	7276 (26.43)	7577 (49.89)	12355 (72.10)	<0.001
**Vegetables, n(%)**					
3 All vegetables	34637 (57.87)	9582 (34.81)	10422 (68.62)	14633 (85.39)	<0.001
**Fruits, n(%)**					
4 All fruits	45274 (75.65)	16610 (60.34)	12669 (83.41)	15995 (93.34)	<0.001
**Dairy foods, n(%)**					
5 Whole milk or derivative equivalents	36943 (61.73)	13006 (47.25)	10191 (67.10)	13746 (80.21)	<0.001
**Protein sources, n(%)**					
6 Beef, lamb, pork	1010 (1.69)	170 (0.62)	236 (1.55)	604 (3.52)	<0.001
7 Chicken, other poultry	2694 (4.50)	655 (2.38)	679 (4.47)	1360 (7.94)	<0.001
8 Eggs	47632 (79.59)	15985 (58.07)	12903 (84.96)	15744 (91.87)	<0.001
9 Fish	14574 (24.35)	3149 (11.44)	3665 (24.13)	7760 (45.28)	<0.001
Legumes					
10 Dry beans, lentils, peas	7798 (13.03)	1758 (6.39)	1960 (12.90)	4080 (23.81)	<0.001
11 Soy foods	59511 (99.46)	27415 (99.59)	15101 (99.43)	16995 (99.17)	<0.001
12 Peanuts or tree nuts	3940 (6.58)	585 (2.13)	788 (5.19)	2567 (14.98)	<0.001
**Added fats, n(%)**					
13 Palm oil, unsaturated oils, dairy fats (included in milk), lard or tallow	10622 (17.75)	2119 (7.70)	2367 (15.58)	6136 (35.81)	<0.001
**Added sugars, n(%)**					
14 All sweeteners	914 (1.53)	597 (2.17)	179 (1.18)	138 (0.81)	<0.001

*The figures in the table mainly indicate the number of participants who reached the recommended food intake. Numbers in parentheses represent% that reached the recommendation within each tertile.

Supplementary Table 4: Average intake of the food components according to EAT-LDP score.

Component	Total intake (g/day), mean (SD)*	Tertiles of EAT-LDP score		
		T1	T2	T3
**Whole grains *and refined grains*, n(%)**				
1 Rice, wheat, corn, and other	166.17 (77.26)	166.56 (75.39)	169.03 (88.47)	163.06 (69.24)
**Tubers and starchy vegetables, n(%)**				
2 Potatoes	59.42 (28.78)	67.66 (36.77)	58.63 (27.40)	54.05 (20.69)
**Vegetables, n(%)**				
3 All vegetables	1296.57 (7202.97)	986.23 (6201.78)	1231.53 (6920.22)	1671.57 (8296.52)
**Fruits, n(%)**				
4 All fruits	331.26 (209.59)	302.31 (208.89)	335.78 (208.63)	361.35 (206.66)
**Dairy foods, n(%)**				
5 Whole milk or derivative equivalents	188.95 (156.11)	192.19 (179.29)	186.86 (150.97)	187.27 (133.17)
**Protein sources, n(%)**				
6 Beef, lamb, pork	98.89 (62.51)	107.23 (67.93)	96.52 (58.10)	85.26 (52.58)
7 Chicken, other poultry	81.42 (33.83)	87.25 (36.46)	80.60 (31.76)	73.35 (29.44)
8 Eggs	77.01 (34.40)	79.02 (35.46)	73.44 (32.35)	70.49 (29.42)
9 Fish	111.28 (53.75)	128.74 (70.33)	111.18 (53.47)	102.04 (39.55)
Legumes				
10 Dry beans, lentils, peas	140.57 (117.47)	185.27 (142.18)	146.71 (119.87)	129.32 (107.64)
11 Soy foods	98.72 (41.77)	103.13 (41.50)	104.25 (48.36)	92.82 (37.30)
12 Peanuts or tree nuts	28.48 (18.55)	23.08 (15.10)	26.45 (17.51)	31.61 (19.63)
**Added fats, n(%)**				
13 Palm oil, unsaturated oils, dairy fats (included in milk), lard or tallow	0.55 (0.34)	0.46 (0.28)	0.54 (0.32)	0.70 (0.40)
**Added sugars, n(%)**				
14 All sweeteners	118.39 (50.65)	109.41 (50.84)	122.50 (50.18)	129.17 (48.14)

Supplementary Table 5: HRs (95% CIs) of T2D incident according to EAT-LDP score group (n=59,351).

	Model 1 [a]	Model 2 [b]	Model 3 [c]	Model 4 [d]
Tertiles of EAT-LDP score				
T1	1.00 (reference)	1.00 (reference)	1.00 (reference)	1.00 (reference)
T2	0.76 (0.68, 0.85)	0.80 (0.71, 0.89)	0.81 (0.71, 0.91)	0.86 (0.76, 0.97)
T3	0.72 (0.65, 0.80)	0.77 (0.69, 0.86)	0.80 (0.71, 0.91)	0.95 (0.84, 1.07)
P for trend	<0.0001	<0.0001	<0.0001	0.219
1-point increment in diet score	0.90 (0.88, 0.93)	0.92 (0.89, 0.95)	0.94 (0.91, 0.97)	0.98 (0.95, 1.02)

Participants were excluded in this sub-analysis if the follow up was less than 3 years.

a Crude model.

b Adjusted for age, sex, Townsend deprivation index, qualifications, and ethnicity.

c Adjusted for age, sex, Townsend deprivation index, qualifications, ethnicity, smoking, drinking, physical activity, energy.

d Adjusted for age, sex, Townsend deprivation index, qualifications, ethnicity, smoking, drinking, physical, energy, BMI and waist circumstance.

Supplementary Table 6: HRs (95% CIs) of T2D incident according to EAT-LDP score group after multiple imputation*.

	**Model 1 [a]**	**Model 2 [b]**	**Model 3 [c]**	**Model 4 [d]**
Tertiles of EAT-LDP score				
T1	1.00 (reference)	1.00 (reference)	1.00 (reference)	1.00 (reference)
T2	0.73 (0.70, 0.78)	0.80(0.75, 0.85)	0.80 (0.74, 0.86)	0.86 (0.80, 0.93)
T3	0.66 (0.63, 0.70)	0.74 (0.70, 0.79)	0.75 (0.70, 0.81)	0.87 (0.80, 0.94)
P for trend	<0.0001	<0.0001	<0.0001	<0.0001
1-point increment in diet score	0.87 (0.85, 0.88)	0.90 (0.88, 0.91)	0.90 (0.89, 0.92)	0.46 (0.92, 0.96)

*: Multiple imputation of missing covariates.

a Crude model.

b Adjusted for age, sex, Townsend deprivation index, qualifications, and ethnicity.

c Adjusted for age, sex, Townsend deprivation index, qualifications, ethnicity, smoking, drinking, physical activity, energy.

d Adjusted for age, sex, Townsend deprivation index, qualifications, ethnicity, smoking, drinking, physical activity, energy, BMI and waist circumference.

Supplementary Table 7: Association between EAT-LDP score and incidence of T2D stratified by different covariates.

	Tertiles of EAT-LDP score			*P* for trend	1 point increment in EAT-LDP score	*P* for interaction
	T1	T2	T3			
**Age**						0.863
<60	1.00 (reference)	0.84 (0.71, 0.99)	0.86 (0.73, 1.02)	0.047	0.92 (0.85, 1.01)	
≥60	1.00 (reference)	0.88 (0.76, 1.02)	0.81 (0.70, 0.94)	0.004	0.94 (0.90, 0.98)	
**Sex**						0.268
Female	1.00 (reference)	0.84 (0.71, 1.00)	0.72 (0.61, 0.86)	<0.0001	0.91 (0.87, 0.96)	
Male	1.00 (reference)	0.85 (0.73, 0.98)	0.88 (0.76, 1.02)	0.046	0.96 (0.92, 1.00)	
**Ethnicity**						0.637
White	1.00 (reference)	0.86 (0.76, 0.97)	0.80 (0.71, 0.90)	<0.0001	0.94 (0.91, 0.97)	
Non-White	1.00 (reference)	0.76 (0.55, 1.05)	0.87 (0.64, 1.19)	0.246	0.96 (0.89, 1.05)	
**Deprivation**						0.653
<median	1.00 (reference)	0.88 (0.75, 1.02)	0.79 (0.68, 0.92)	0.002	0.94 (0.90, 0.98)	
≥median	1.00 (reference)	0.81 (0.69, 0.95)	0.82 (0.70, 0.96)	0.005	0.94 (0.90, 0.98)	
**Education attainment**						0.748
College or university degree	1.00 (reference)	0.92 (0.75, 1.11)	0.83 (0.69, 1.00)	0.055	0.93 (0.88, 0.98)	
Professional qualifications	1.00 (reference)	0.69 (0.43, 1.11)	0.82 (0.52, 1.28)	0.310	0.97 (0.85, 1.11)	
Others	1.00 (reference)	0.84 (0.73, 0.97)	0.79 (0.69, 0.91)	0.001	0.94 (0.91, 0.98)	
**Smoking status**						0.673
Never	1.00 (reference)	0.82 (0.69, 0.96)	0.81 (0.69, 0.95)	0.006	0.94 (0.90, 0.98)	
Previous	1.00 (reference)	0.90 (0.76, 1.06)	0.77 (0.65, 0.92)	0.003	0.92 (0.88, 0.97)	
Current	1.00 (reference)	0.85 (0.58,1.23)	0.97 (0.66, 1.44)	0.706	1.01 (0.92, 1.12)	
**Drinking status**						0.418
Never	1.00 (reference)	0.93 (0.61, 1.41)	0.54 (0.32, 0.89)	0.021	0.85(0.75, 0.96)	
Previous	1.00 (reference)	0.76 (0.46, 1.24)	1.05 (0.68, 1.62)	0.997	1.02 (0.90, 1.15)	
Current	1.00 (reference)	0.84 (0.75, 0.95)	0.81 (0.72, 0.91)	<0.0001	0.94 (0.91, 0.97)	

Supplementary Figure 1: Distribution of the EAT-LDP score in the 59,849 participants.

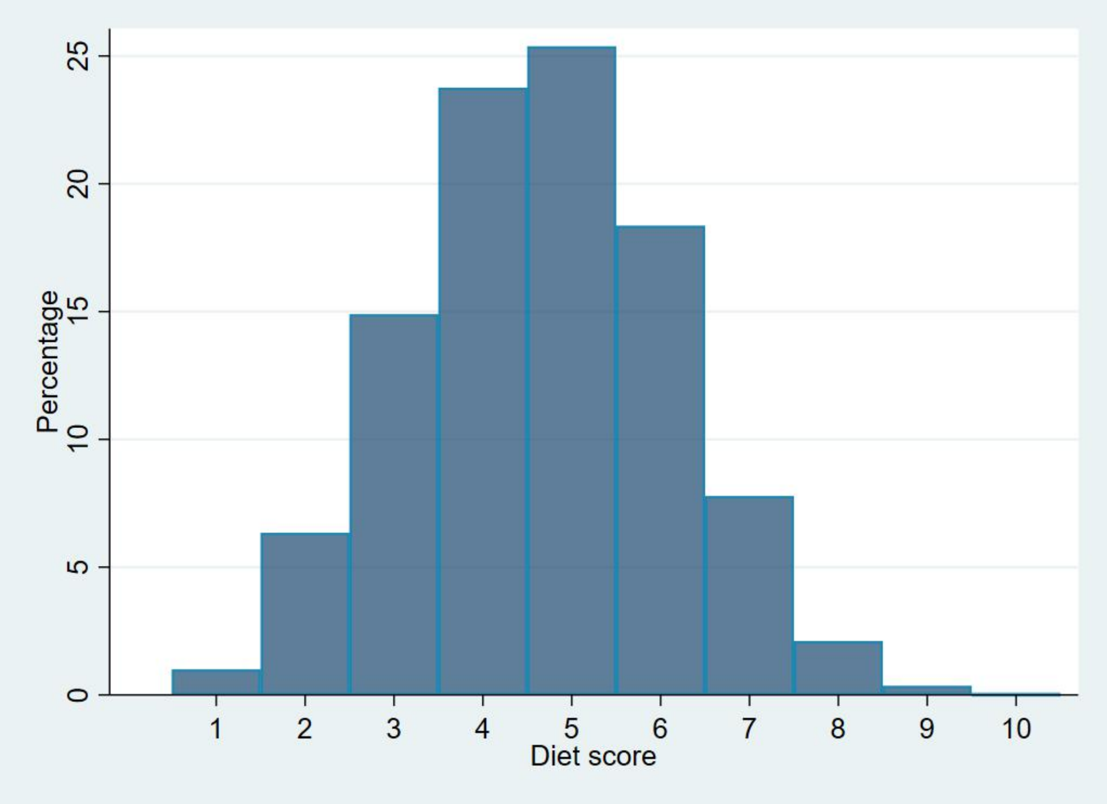

Supplementary Figure 2: Association between adherence of specific food recommendations and incident T2D.

Component	Recommended amount based on the EAT-LDP	NO. of participants meeting the recommendations	HR (95% CI)*	*P* value
1 Rice, wheat, corn, and other	≤ 464g/day	45100	1.23 (0.46, 3.30)	0.675
2 Potatoes and cassava	≤100g/day	27208	0.83 (0.75, 0.91)	<0.0001
3 All vegetables	≥200g/day	34637	0.88 (0.81, 0.96)	<0.0001
4 All fruits	≥100g/day	45274	0.87 (0.79, 0.95)	0.002
5 Dairy foods	≤500g/day	36943	0.78 (0.62, 0.97)	0.027
6 Beef, lamb, pork	≤28g/day	1010	0.60 (0.40, 0.90)	0.013
7 Chicken, other poultry	≤58g/day	2694	1.11 (0.92, 1.36)	0.27
8 Eggs	≤25g/day	47632	0.84 (0.75, 0.93)	0.001
9 Fish	≤100g/day	14574	0.92 (0.84, 1.02)	0.109
10 Dry beans, lentils, peas	≤100g/day	7798	0.98 (0.86, 1.12)	0.790
11 Soy foods	≤50g/day	59511	1.03 (0.27, 3.85)	0.971
12 Peanuts or tree nuts	≥25g/day	3940	0.94 (0.79, 1.12)	0.492
13 Added fats	ratio: 0.8**	10622	0.94 (0.85, 1.04)	0.239
14 Added sugars	≤50g/day	914	1.85 (1.45, 2.37)	<0.0001

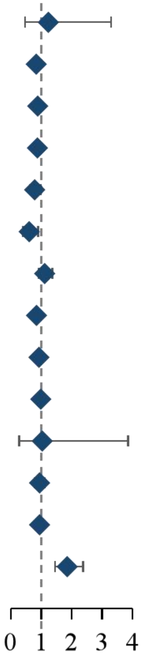

* HRs for adherence vs. non-adherence participants, and were adjusted for age, sex, Townsend deprivation index, qualifications, ethnicity, smoking, drinking, physical activity, and energy.

** Ratio of 0.8 for unsaturated: saturated fat intake.
